# Resistance of Resin-Bonded Ceramic Endocrowns of Different Intracoronal Depths

**DOI:** 10.3290/j.ohpd.c_1817

**Published:** 2025-01-22

**Authors:** Hatem Alqarni, Majd Qadoumi, Nouf AlShehri, Norah AlNowaiser, Razan Alaqeely, Abdulaziz A. AlHelal, Mohammed Alrabiah, Abdulmonem Alshihri, Hussain Alsayed

**Affiliations:** a; b Hatem Alqarni Assistant Professor, Restorative and Prosthetic Dental Science Department, College of Dentistry, King Saud Bin Abdulaziz University for Health Sciences, Riyadh, Saudi Arabia; King Abdullah International Medical Research Center, Ministry of National Guard Health Affairs, Riyadh, Saudi Arabia. Conceptualisation, methodology, investigation, writing (original draft), writing (review and editing), visualisation.; c Majd Qadoumi Intern, Dental University Hospital, King Saud University, Riyadh, Saudi Arabia. Conceptualisation, methodology, investigation, writing (original draft), writing (review and editing).; d Nouf AlShehri Intern, Dental University Hospital, King Saud University, Riyadh, Saudi Arabia. Conceptualisation, methodology, investigation, writing (original draft), writing (review and editing).; e Norah AlNowaiser Intern, Dental University Hospital, King Saud University, Riyadh, Saudi Arabia. Conceptualisation, methodology, investigation, writing (original draft), writing (review and editing).; f Razan Alaqeely Assistant Professor, Department of Periodontics and Community Dentistry, College of Dentistry, King Saud University, Riyadh, Saudi Arabia. Investigation, writing (original draft), writing (review and editing).; g; h Abdulaziz A. AlHelal Associate Professor, Department of Prosthetic Dental Sciences, College of Dentistry, King Saud University, Riyadh, Saudi Arabia. Investigation, data curation, writing (review and editing).; i Mohammed Alrabiah Associate Professor, Department of Prosthetic Dental Sciences, College of Dentistry, King Saud University, Riyadh, Saudi Arabia. Investigation, data curation, writing (review and editing).; j Abdulmonem Alshihri Associate Professor, Department of Prosthetic Dental Sciences, College of Dentistry, King Saud University, Riyadh, Saudi Arabia. Data curation, writing (review and editing).; k Hussain Alsayed Associate Professor, Department of Prosthetic Dental Sciences, College of Dentistry, King Saud University, Riyadh, Saudi Arabia. Conceptualisation, methodology, investigation, writing (original draft), writing (review and editing), visualisation.

**Keywords:** endocrown, fracture, intracoronal depth, resistance

## Abstract

**Purpose:**

This *in-vitro* study was conducted to assess the fracture resistance of resin-bonded ceramic endocrowns with different designs at varying intracoronal depths.

**Materials and Methods:**

Forty-eight (n = 48) extracted mandibular first molar teeth were randomly divided into four groups (n = 12). In the control group, the specimens remained untreated. Whereas the specimens in the test groups A, B, and C were decapitated 2 mm above the cementoenamel junction (CEJ) and endodontically treated. The test groups were prepared with a butt-joint design in a standardised manner with varying intracoronal depths. Groups A, B, and C were prepared to receive lithium disilicate endocrown with intracoronal cores at 0 mm, 2 mm, and 4 mm, respectively. Crowns were fabricated as a non-anatomical design with a thickness of 3 mm. After ceramic bonding procedures, specimens underwent thermocyclic ageing prior to the fracture resistance test. Specimens were loaded at a 15-degree angle using the Universal Testing Machine and the failure modes were observed. One-way analysis of variance (ANOVA) and Chi-square were utilised for data statistical analyses.

**Results:**

Significant statistical results in fracture resistance tests were found in all experimental groups. The highest load was found in group B, followed by group C, and lastly group A (P < 0.05). Although endocrowns with no extension had the lowest fracture resistance, they showed a favourable cohesive failure with statistically no significant difference from the control group.

**Conclusion:**

In bonded ceramic endocrowns, the fracture resistance is not newcessarily proportional to the intracanal depth. The intrcoronal cores of 4 mm did not show the highest fracture resistance, and their mode of failure was catastrophic compared to endocrowns with no intracoronal extensions.

It is a daily challenge for a dentist to maintain and preserve the tooth structure, especially the enamel. The enamel ensures mechanical stability, preserves the integrity of the restoration, and increases the adhesive surfaces that greatly impact the effectiveness of the longevity and durability success.^27^ It is necessary to understand that endodontically treated teeth differ from vital teeth. Major changes following treatment include altered tissue physical characteristics, loss of tooth structure, and possibly discolouration. Clinical studies reported the strong impact of those changes on the long-term survival of endodontically treated teeth.^13^

There have been several reported cases where teeth catastrophically fracture, resulting in the removal of the tooth, especially in the case of multirooted teeth. Therefore, preserving the tooth structure is a prime factor in improving biomechanical and chemical bonding.^27^ Dental materials should be selected to achieve optimal mechanical and functional properties, aesthetics, and coronal seal.^4^ Restorations of endodontically treated teeth are designed to protect the remaining tooth from fracture, prevent reinfection of the root canal system, and replace the missing tooth structure.^13^

The key objective is to improve the fracture resistance and reduce the loss of structural integrity of the endodontically treated teeth by using a conservative cavity preparation. Trauma from occlusal or caries damage and loss of micro-spaces between the tooth and the restoration can all lead to the loss of structural integrity. Studies provide a strong indication that marginal ridge loss is the major cause of reduction and durability in endodontically treated teeth, whereas some studies have found that cavity preparation and root canal preparation triggers a strong change in tooth structure loss, resulting in variability of dentine brittleness.^26^

Controversies in the literature exist on which material or procedure will ideally restore the endodontically treated teeth.^11^ The typical procedure to restore endodontically treated teeth is by using adhesive procedures and placing crowns with a good amount of ferrule.^15^ Prefabricated metal posts are a quick and simple treatment option, but they do not consider the individual shape of the root canal, which reduces their adaptation property. The preparation of a post space increases the risk of accidental mishaps such as root perforation.^14^ Cast metal post-buildups are considered the most appropriate alternative for wide and uneven canals as they are obtained based on a mould made directly from the root cavity to achieve intimate adaptation between the dental canal and the post system. However, root fractures can occur due to the high rigidity of metallic alloys that are widely used for casting posts compared to dentine.^14^

Later, fibre posts were introduced as customisable post-systems made from materials that have very similar elastic properties to those of natural teeth. The significant discrepancy between the rigidity of intraradicular systems and dental tissues in the restorative materials and tooth interfaces will generate significant stress concentration. This has contributed to the decrease in the likelihood of catastrophic fracturing, which manifested as debonding of the post.^14^

Based on microstructure, dental ceramics could be classified into three groups: predominantly glass ceramics, particle-filled glasses, and polycrystalline ceramics. Dental ceramics that mimic the enamel, dentine, and their optical properties are predominantly glass ceramics. They are made of three networks of atoms with no clear pattern of the spacing between the nearest or the next nearest atom, which is called an ‘amorphous form’. In dentistry, glass is principally derived from an amine minerals group that is called feldspar which is based on silica (silicon oxide) and alumina (aluminium oxide). Hence, feldspathic ceramics belong to a family that is called aluminosilicate glasses.^5,29^ The traditional feldspathic ceramics could be described as the most aesthetic translucent material and are typically used for veneering porcelains, as frameworks of metal ceramics, or for bonded laminate veneers.^21^

To control the optical effects such as colour, opacity, and opalescence along with improving the mechanical properties, filler particles were added to the composition of the base glass. Usually, these fillers are crystalline but can also be of higher melting glass particles.^29^ Leucite-reinforced lithium disilicate ceramics and zirconia-reinforced lithium silicate are composed of the crystalline phase, which is incorporated in a glassy matrix and is commonly used due to its good shade and flexural strength. However, one of its main disadvantages is the 15–20% polymerisation contraction that reduces the density between the restoration and the tooth in the second sintering process.^23^

Polycrystalline ceramics, which are also called (oxide ceramics) such as zirconia (zirconium oxide), are characterised by very good mechanical properties that are significantly greater than silica-based ceramics.^21^ This material is characterised by a monocrystalline homogeneity that is dense and possesses reduced thermal conductivity, reduced corrosion potential, and good radiopacity.^28^ The first generation of zirconia had limited translucency and because of that, its use was limited for frameworks and coping, which then had to be veneered by feldspathic veneering porcelain. However, the latest generation offered greater light transmission. For anterior teeth, the high-translucent multilayer pre-shaded zirconia offered more aesthetic options for treatment than have ever been applied.^21^

Modern approaches focus on achieving more conservative cavity designs by basically providing enough access for completely removing the carious tissue. Subsequent restorative procedures rely mainly on the effectiveness of the bonding of the adhesive material.^6^ In the last decade, the use of indirect restorations has been more often which is triggered by the advances in the development of material and manufacturing technologies. Nowadays, bonded restorations are considered an integral part of the field of minimally invasive dentistry because of the advances in the technology of adhesion. Besides the restoration strength for long-term clinical success, the adhesion of the luting cement to the two substrates (ie, the material of the indirect restoration and the dental tissues) is also important.^25^

Recently, there has been a change in treatment options towards further conservative modalities with the reported developments in adhesive dentistry, doubting the necessity of conventional posts and cores. Ceramic restorations such as endocrowns have indeed been developed as alternative approaches to restoring endodontically treated teeth based on the quality of the existing surface of the tooth structure.^3^ The endocrown makes the use of surface available in the pulp chamber to ensure the restoration is durable and maintained by adhesive bonding for retention.^18^

The term ‘endocrown’ initially introduced by Pissis in 1995,^22^ and later described by Bindl and Mormann in 1999,^3^ is an adhesive, monoblock restoration comprised of two parts: a crown and a retainer that is anchored inside the pulp chamber of a devitalised tooth.^24^ The anchorage to the internal aspect of the pulpal chamber is what provides adequate micromechanical retention between the endocrown and the remaining tooth structure with the usage of adhesive cement, which provides micromechanical retention.^24,31^ Hence, endocrowns have been proposed as a conservative, minimally invasive treatment approach. Endocrowns can be helpful in cases of severely curved or calcified canals; where the placement of a traditional post and core could be quite difficult.^2^ Some recommended guidelines for an endocrown preparation generally include a cuspal reduction of approximately 2–3 mm, circular equigingival butt-joint margin of 90 degrees, a smooth, and relatively flattened pulpal floor, smooth internal transitions, an internal taper of 6 degrees for the pulpal chamber, and supragingival margins when possible.^3,9,16^ In comparison to the conventional preparation designs that utilise posts, the anchorage of endocrowns is derived from two components: the pulp chamber and the margins of the cavity. Thereby, attaining both macro- and micromechanical retention, provided by the walls of the pulp chamber and the adhesive cement. Preparations of teeth receiving endocrowns require less sound tissue removal and less chairside time, when compared to other methods. Studies have also shown that upon mastication, heavy loads and stresses are better scattered in teeth restored with endocrowns. Endocrowns can be used to restore teeth that are comprised of short and dilacerated roots, for example. In addition, in a clinical situation where minimal interocclusal space is present, endocrowns would be considered as the appropriate method of restoration. In terms of the presence of a ferrule, a notable controversy is present. Fasbinder et al suggests that the ferrule effect should be avoided when preparing the tooth for an endocrown,^7^ whereas Einhorn et al states that the significance of a ferrule effect has not been thoroughly studied.^8^

Moreover, as dentistry shifts towards a more convenient, innovative, and technology-oriented end, endocrowns have become available with the introduction of computer-aided design and manufacturing (CAD/CAM) technology. The restoration can be developed through digital impressions made by a hand-held scanner with a built-in camera (CAD). This digital data is then converted to produce a restoration from a block through a process called ‘milling’ (CAM).^9^ Therefore, the possible errors during the milling process can be avoided or controlled.^6^ The benefits of the CAD/CAM system include the facilitation of chairside designing and the development of various restorations.^12^ In addition, various studies have shown that with CAD/CAM technology, a standard of appropriate strength, marginal fit, and aesthetics is attainable.^18^

To the author’s knowledge, current literature lacks specific recommendations in terms of intracoronal depths of the endocrown. Therefore, the aim and purpose of this study are to assess and investigate the fracture resistance of endocrowns at varying intracoronal depths.

## MATERIALS AND METHODS

### Eligibility Criteria

The Institutional Review Board approval was granted to conduct this in-vitro study. The inclusion criteria were mandibular first molars with mature apices, absence of cracks, non-carious, and absence of any other defects. Teeth were selected with similar buccolingual (BL) and mesiodistal (MD) dimensions (10.24 mm and 10.56 mm, respectively), as measured with a digital calliper allowing a maximum deviation of 10% from the determined mean.^30^ Teeth were cleaned using hand scaling followed by using a rubber cup and pumice slurry.

### Sampling and Grouping

**Fig 1:**
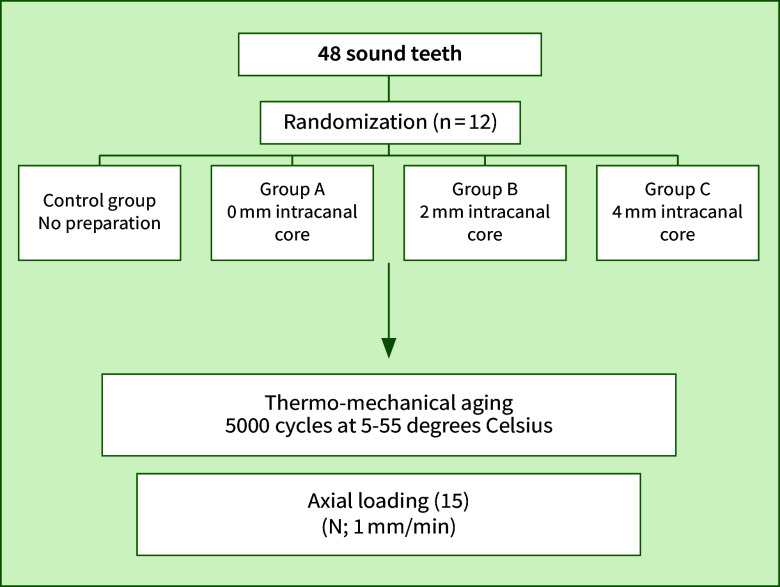
Overview of the study groups & flow chart showing the experimental sequence. 0, 2, and 4 mm intracanal core of endocrowns.

The sample teeth were randomised using an online randomisation generator and then divided into 4 groups of 12 specimens each (Fig 1). The control group, which are sound teeth without any preparation or restoration. Group A represent teeth that were prepared with a butt-joint preparation design and received an endocrown with 0 mm intracanal core. Group B, which represents teeth that were prepared with a butt-joint preparation design and received endocrown with a 2 mm intracanal core. Group C represent teeth that were prepared with a butt-joint preparation design and received an endocrown with a 4 mm intracanal core. All teeth in the experimental groups were decapitated using a coarse diamond wheel bur (5909 FG; Komet Dental, Lemgo, Germany) 2 mm above the cementoenamel junction. All the endodontically treated teeth were prepared using a K9 milling machine (Kavo Fräsgerät Ewl Typ 990; mit Modelltisch & Zubehör, Germany) to standardise the preparation dimensions.

### Endodontic Protocol

Experimental groups received endodontic treatment. Endodontic procedures (access cavity preparation, instrumentation, and obturation) were performed by an endodontist. Standard straight line access cavity preparation with surgical carbide round burs (Dentsply Tulsa Dental Specialties, Tulsa, Oklahoma, USA) were used starting with a size of 4 mm in diameter and a 25 mm shank length, followed by a size of 1.4 mm diameter and a 25 mm shank length. Round-point-tapered burs (Dentsply Tulsa Dental specialties) were used in high-speed contra-angle handpieces (Sirius: micro-mega; Besancon, France). The pulp tissues were removed with barbed broaches (No.33 colour-coded, plastic handle, (Dentsply Tulsa Dental Specialties).

The working length was determined by visualising the tip of size #10 K file (Dentsply Tulsa Dental Specialties) exited from the apical foramen of the root. The file length was then reduced by 0.5 mm from this measurement. Flowable resin composite (Ivoclar Vivadent, Heliomolar Flow Dental Flowable Composite) was used to seal the apices and a glide path was established up to size 25 K file. The teeth were attached to the dental surveyor’s analysing rod (Ney Dental, Bloomfield, CT, USA) using a light-curing composite with a long tooth axis parallel to the analysing rod to ensure that the tooth orientation for all specimens is consistent. The teeth were then mounted in moulds and the brass ring was filled with ortho resin (Interacryl ortho, Interdent, Celje, Slovenia). The attached tooth to the analysing rod was lowered to the centre of the mould until the ortho resin embedded the tooth to a level of 2 mm below the CEJ to complete the tooth stabilisation.

**Fig 2a and b:**
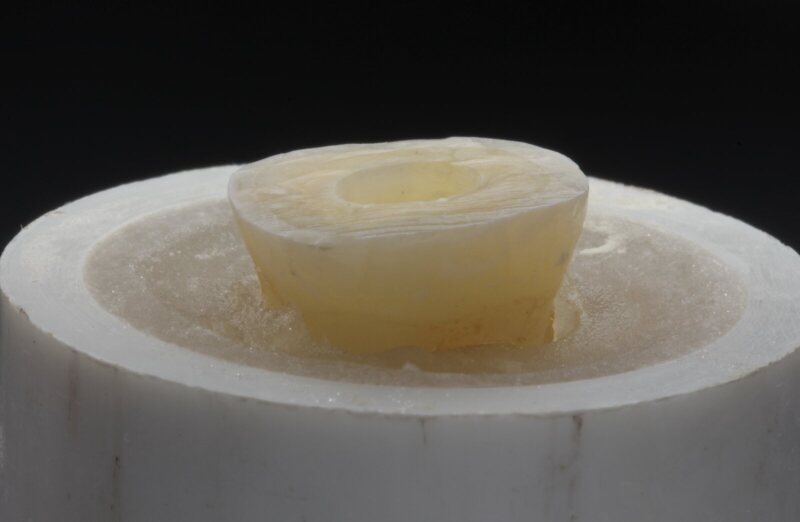

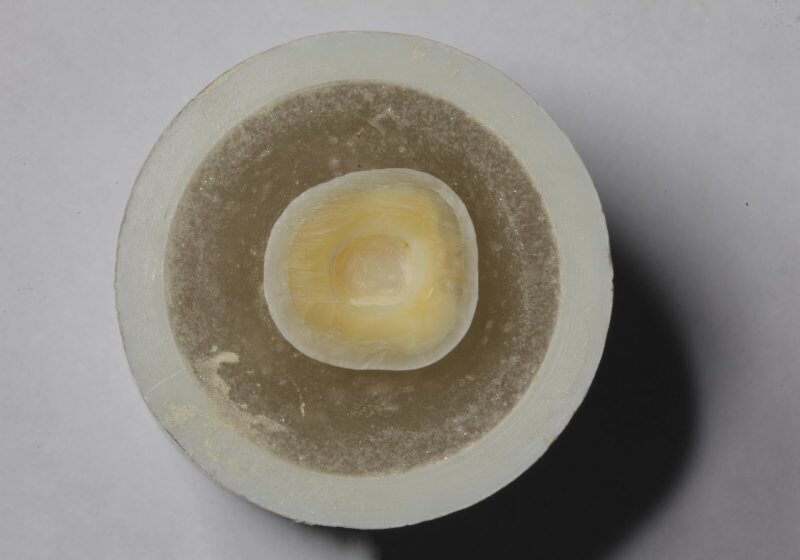
The sample of intracoronal depths of 2 mm before endocrown fabrication. Lateral (a). Occlusal views (b) .

The preparation of the root canal system (cleaning and shaping) was done by using machine-driven rotary files ProTaper following the sequence S1, S2, F1, F2, F3 (Dentsply Maillefer, Ballaigues, Switzerland) using EDTA (ethylenediaminetetraacetic acid solution) (Pulpdent, Watertown, MA, USA) to remove the smear layer for 30 s. After each episode of root canal instrumentation, the root canals were irrigated with 15 cc of 2.26% sodium hypochlorite solution (NaOcl), by using a 27-gauge endodontic needle. The canals were then dried by using paper points (Dr. Wild & Co, Basel, Switzerland) and obturated using matching Gutta-Percha points (Roeko, Langenau, Germany) by the lateral cold condensation technique using the AH 26 sealer (Dentsply De-Trey, Konstanz, Germany). The obturation material was cut at the orifice of the canal with the use of system B (Analytic, Sybron Dental Specialties, Orange, CA, USA) according to the manufacturer’s instructions. Hence, providing a standardised filling procedure. After the obturation of all samples, standard preparations of the teeth were done by a prosthodontist using a wheel diamond bur to ensure that the specimens were flat occlusally, followed by a tapered diamond bur on a high-speed handpiece to ensure that no undercuts were present within the cavity. Flowable resin composite (Ivoclar Vivadent, Heliomolar Flow Dental Flowable Composite) was then used to fill the cavities based on the measurements of 0, 2, and 4 mm. A periodontal probe was used to take five measurements in each cavity – mesiobuccal, distobuccal, mesiolingual, distolingual, and central – to confirm a flat pulpal floor with the correct, standard measurements (Fig 2).

### Wax-up and Pressing

Endocrown restorations were initially waxed-up and designed to be non-anatomical with a thickness of 3 mm. Investment of crowns was done using phosphate-bonded investment (Ivoclar Vivadent IPS PressVEST) followed by firing for approximately two hours. Crowns were then pressed (IPS E.max press Ingots, low translucency, shade A1) and finished using appropriate burs to ensure proper smoothening.

### Try-in and Cementation

**Fig 3:**
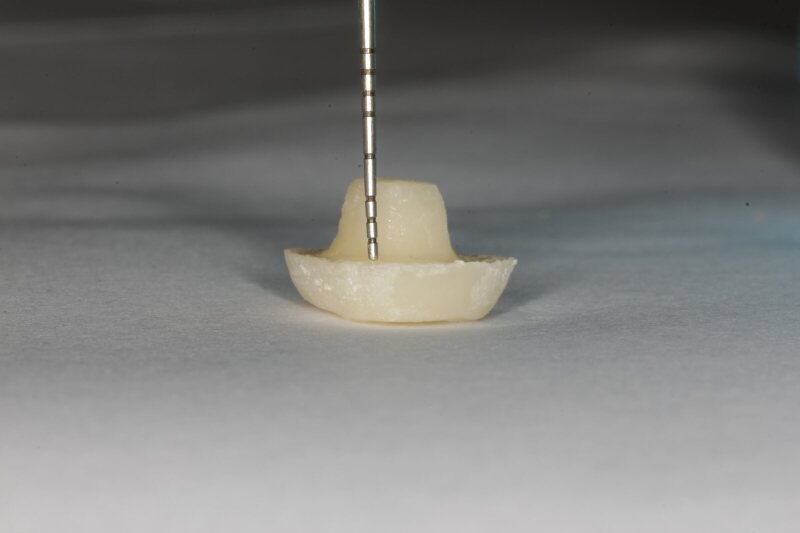
Sample of 4 mm intracoronal depths before cementation.

Endocrowns for groups were tried-in for appropriate and complete fitting of restoration. Endocrowns with inaccuracies were redone. The endocrown was etched with 10% hydrofluoric acid (Ivoclar Vivadent Inc) for 20 s and then rinsed with water. Finally, the endocrown was cemented with self-etch and self-adhesive cement (Kerr Maxcem Elite Universal Resin Cement). Upon seating the endocrown on the tooth, the light-curing unit (Bluephase, Ivoclar Vivadent, Schaan/Liechtenstein) was used to ensure the complete setting of the cement (Fig 3).

### Thermocycling

After cementation of the restorations, the specimens are subjected to approximately 5,000 cycles of thermocycling at 5–55°C. The thermocycling process is made possible using the SD Mechatronik (Miesbacher Str. 34. 83620 Feldkirchen-Westerham Thermocycler, Germany).^1^ The thermocycling process allows the exposure of the specimens to varying temperature ranges. This, in turn, may produce consequences owing to the different coefficients of thermal expansion.

### Fracture Resistance Test

**Fig 4a and b:**
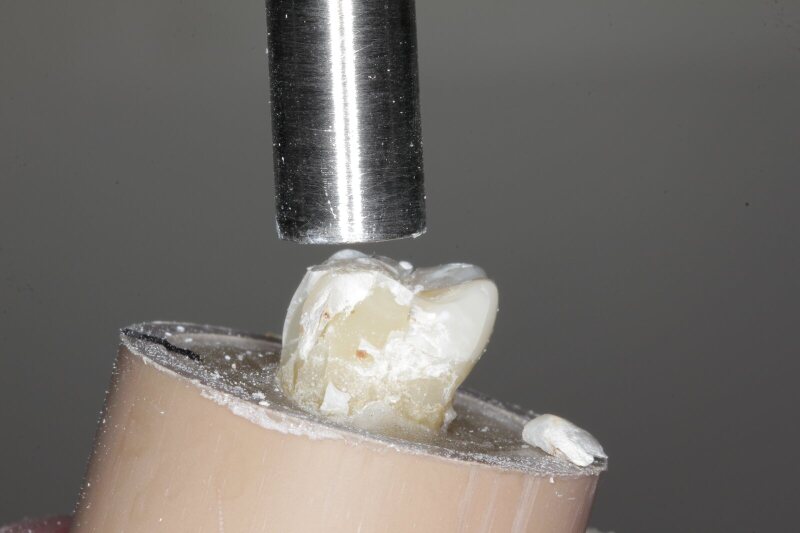

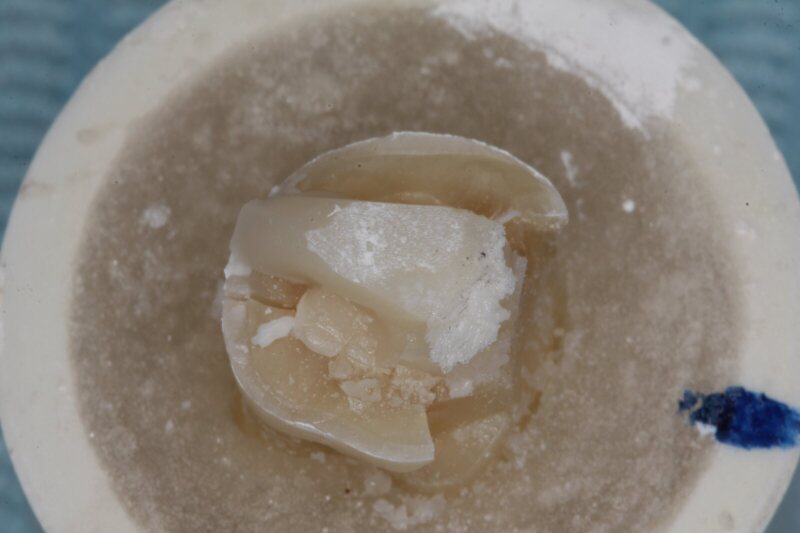
Sample mounted at 15-degree angle for fracture resistance test (a). Catastrophic fracture of the sample of 4 mm intracoronal depths after resistance load (b).

The teeth were stored for 24 h before initiating the fracture resistance test. A pilot study was conducted prior in which one sample from groups A and B mm were taken. Specimens were mounted in a jig that allowed loading at the central fossa with a buccal orientation in the axio-occlusal line at a 15-degree angle (Fig 4a). The Instron 5965 Universal Testing Machine (Instron, Norwood MA, United States of America) was used for this test by delivering a compressive load directed at the central fossa at a speed of 0.5 mm min^–1^ until failure occurred. This load was measured in Newtons (N). The endocrown of the sample in group 0 was debonded without fracture of the tooth, whereas the endocrowns from the samples in groups B and C were fractured at both the crown and root and the crown, respectively. Therefore, failures are subcategorised as favourable and unfavourable. Favourable failures, overall, are identified as repairable, whereas failures deemed unfavourable include vertical root fractures and unrepairable failures. The fracture resistance test will take place in the same manner for the remaining samples.

### Statistical Analysis

Sample size determination was set at a level of significance of 0.05 with the effect size of 0.55 at a power of 0.85, the total sample size should be at least 48, 12 samples in each group. Data will be analysed using IBM/SPSS version 25 statistical software. Normality testing will be used utilising the Shapiro test. If the normality is satisfied, the Levene test also will be used to test the homogeneity of variance. One-way ANOVA will then be used to compare the groups by their average load of MPa. If the P-value is significant, then Games–Howell will be utilised as a multiple comparison test (MCT). Finally, the Chi-square test will be used to study the association between groups of favourable and unfavourable results (0 unfavourable, 1 favourable).

## RESULTS

The mean average of the fracture resistance in the four groups is shown in Figure 1. Endocrowns made with 2 mm intracoronal depths (group B) showed a higher fracture resistance among all the groups. The endocrowns with 4 mm intracoronal depths (group C) come next, followed by the control group, and lastly, the endocrowns with no intracoronal extensions. The means, standard deviations, and 95% confidence interval levels are displayed in Table 1. One-way ANOVA shows a significant difference between groups in the fracture resistance test (P <0.05). The Games–Howell was utilised for multiple comparisons of the fracture resistance of the different intracoronal endocrown indicated that there was a significantly higher value (P <0.05) of fracture resistance of 2 mm intracoronal depths of endocrowns (group B). Also, there was no significant difference between groups A mm and C mm as shown in Table 2.

**Table 1:** Mean of fracture resistance is shown with the standard deviations of each group. One-way ANOVA shows a significant difference between groups in the fracture resistance testy

Group	N	Mean	Std. Deviation	P-value	95% Confidence Interval for mean
Lower bound	Upper bound
Group A mm	12	53.387	15.799	0.007*	43.348	63.425
Group B mm	12	84.750	25.489	68.555	100.945
Group C mm	12	81.919	37.514	58.084	105.755
Control group	12	59.381	26.898	42.291	76.471

**Table 2:** Games–Howell multiple comparison test shows a significant difference between group A and group B

Group	Group 0 mm	Group 2 mm	Group 4 mm	Group control
Group A mm	1	0.009*	0.115	0.909
Group B mm	0.009*	1	0.996	0.996
Group C mm	0.115	0.996	1	0.354
Group control	0.909	0.996	0.354	1


The Games–Howell was utilised for multiple comparisons of the fracture resistance of the different intracoronal endocrown indicated that there was a significantly higher value (P <0.05) of fracture resistance of 2 mm intracoronal depths of endocrowns (group B). Also, there was no significant difference between groups A mm and C mm as shown in Table 2.

All the specimens in group A exhibited a favourable cohesive failure of the endocrowns, with no catastrophic fractures of natural teeth. However, group B and C both have unfavourable adhesive failures where both the endocrowns and the natural teeth were catastrophically fractured (Fig 4b). Furthermore, the Chi-square test for the mode of failure revealed statistically significant results among all test groups (Table 3).

**Table 3:** Chi-square test for the mode of failure. Note statistically significant results among all test groups

Factor	Level	Stat	Favourable mode	Total	Ch-sq P-value
Unfavourable	Favourable
Group	Group A mm	Count	0	12	12	0.000
% within group	0.0%	100.0%	100.0%
Group B mm	Count	12	0	12
% within group	100.0%	0.0%	100.0%
Group C mm	Count	12	0	12
% within group	100.0%	0.0%	100.0%
Group control	Count	8	4	12
% within group	66.7%	33.3%	100.0%


## DISCUSSION

In this study, a representation of the heavily destructed tooth structure was made to investigate an alternative to the conventional crown and the absence of absolute necessity of the ferrule effect presence. In such clinical situations, utilising the recent development of adhesive dentistry is considered a conservative, valuable approach. The high fracture loads in the experimental groups can be explained by increasing the surface area of a cemented endocrown to the endodontically treated teeth.^20^ Subjecting the specimens to thermocyclic ageing and mechanical loads is a widely accepted method of laboratory testing. Exposure of the luting agent to interchangeable temperatures helps resemble a clinical situation by subjecting the hybrid layer to hydrolysis and leakage due to differences in the coefficient of thermal expansion between different dental materials and the tooth structure.^10^

Several studies reported different aspects of endocrown designs and usages. Shin et al investigated the marginal and internal discrepancies of endocrowns with different cavity depths and found out that endocrowns with a 4 mm intracoronal depths (group C) showed higher discrepancies and internal volume than 2 mm cavity depth endocrowns (group B).^26^

To the authors’ knowledge, no study examined the fracture resistance of endocrowns with different intracoronal depths after ageing. Another *in-vitro* study done by Turkistani et al showed the fracture resistance of various endocrown heights of 3, 4.5, and 6 mm measured about 2 mm above cementoenamel junction were studied. It was found that the 3 mm crown had the greatest fracture resistance among all other groups, with 90% of the samples having root fractures.^31^ Contrary to this study, the crown thickness was determined to be 3 mm with all the experimental groups. Since the average height of a human mandibular molar is 7 mm measured from cementoenamel junction, the endocrowns were designed with intracoronal extensions to gain better mechanical properties and to override the limitation of short clinical crowns.^19^ Utilising 2 mm intracoronal extensions of the pulp chamber provided greater fracture resistance in comparison to 0 mm and 4 mm depths of endocrown extensions. Unfortunately, failure modes for groups B and C (2 mm and 4 mm intracoronal depths, respectively) were adhesive failures that ended with catastrophic fractures of all the experimental teeth. Therefore, it might be advantageous in certain clinical situations to rehabilitate the endodontically treated teeth with 0 mm endocrown to avoid unfavourable tooth fractures as it was revealed in this study.

A limitation of the current study is the use of one restorative material. Different results might be achieved with the use of multiple restorative materials and/or fabrication techniques such as CAD/CAM. Therefore, further studies are needed to investigate the effect of intracoronal depths in relation to other variables, such as material difference and the method of fabrication. The author suggests that future studies should investigate the effects of various preparation designs on the fracture resistance of endocrowns, such as the presence of a ferrule rather than a butt-joint design. Another limitation includes the presence of one type of load. Future research should assess and examine the cyclic resistance of endocrowns induced by cyclic loading, instead of static. This can be accomplished with the help of a chewing simulator, for instance.

## CONCLUSIONS

The presented study helps the clinician to understand different aspects of designing crown extensions of the endocrowns. The presence of an adequate intracoronal space facilitates the use of endocrowns with just 2 mm intracoronal extensions to have the maximum fracture resistance load. In a clinical situation where the tooth is too short, endocrowns with no intracoronal extensions can be a viable treatment option with possibly the least catastrophic complications.
